# Delayed diagnosis of acromegaly in a patient with focal segmental Glomerulosclerosis: a rare case report and literature review

**DOI:** 10.1186/s12882-019-1626-1

**Published:** 2019-11-27

**Authors:** Jia Zheng, Zhao Cui, Ji-cheng Lv, Hong-zhou Duan, Su-xia Wang, Jun-qing Zhang, Fu-de Zhou, Xiao-hui Guo, Ming-hui Zhao

**Affiliations:** 1 Department of Endocrinology, Peking University First Hospital, Beijing, 100034 China; 2 Renal Division, Department of Medicine, Peking University First Hospital; Institute of Nephrology, Peking University; Key Laboratory of Renal Disease, Ministry of Health of China; Key Laboratory of CKD Prevention and Treatment, Ministry of Education of China, Beijing, 100034 China; 3 Department of Neurosurgery, Peking University First Hospital, Beijing, 100034 China; 4 Electron microscopy laboratory, Peking University First Hospital, Beijing, 100034 China; 5 Peking-Tsinghua Center for Life Sciences, Beijing, 100871 China

**Keywords:** Focal segmental glomerulosclerosis, Acromegaly, Treatment, Growth hormone, Pituitary adenoma

## Abstract

**Background:**

Experimental studies have demonstrated that hypersecretion of growth hormone (GH) is associated with development of glomerular sclerosis. However, clinical case of such condition is very rare. Here we presented a case of focal segmental glomerulosclerosis (FSGS) associated with acromegaly.

**Case presentation:**

A 63-year-old man was diagnosed as nephrotic syndrome with minimal change disease for 2 years. Prednisone 1 mg/kg/day for 9 months led to no response. After admission, the second kidney biopsy indicated FSGS (NOS variant). On admission, his acromegalic features were noticed and he complained with a 20-year history of soft tissue swelling of hands and feet. Serum GH and insulin-like growth factor 1 (IGF-1) concentrations were both elevated significantly. An oral glucose tolerance test showed inadequate suppression of serum GH. The presence of a pituitary macroadenoma with a diameter of 1.4 cm by MRI confirmed the diagnosis of acromegaly. Then, the tumor was subtotally removed by trans-sphenoidal surgery. Partial remission of proteinuria was achieved 3 months after surgery and maintained during follow-up, with gradual reduce of corticosteroid.

**Conclusions:**

This rare case suggested that the hypersecretion of GH may participate, at least in part, in FSGS development and progression. Early diagnosis and treatment of acromegaly is beneficial.

## Background

Focal segmental glomerulosclerosis (FSGS) is an important cause of nephrotic syndrome in children and adolescents, and may lead to end-stage renal disease [1, 2]. Primary FSGS is presumably caused by generalized podocytes injury and depletion. Secondary FSGS may result from various diseases, including maladaptive from functioning nephrons reduction or abnormal stress, drug-induced FSGS, and virus-associated FSGS [3]. Glomerular hypertrophy and hyperfiltration are common causes of the development of secondary FSGS [4].

Acromegaly is a neuroendocrine disease characterized with acral enlargement, growth hormone (GH) hypersecretion, increased levels of insulin-like growth factor 1 (IGF-1), and most the result of a pituitary tumor producing GH [5]. It indicates that glomerular hypertrophy and hyperfiltration is present in patients with acromegaly [6]. In a chronically expressing GH mice model, progressive glomerulosclerosis with mesangial cell proliferation and immune deposits were observed [7]. However, it is very rare in clinical case with glomerular lesions associated with GH excessive secretion. Patients of acromegaly who are presented with FSGS is also scarce.

Herein, we reported a rare case of delayed diagnosis of acromegaly with a giant GH-producing pituitary tumor, who was concurrent with nephrotic syndrome of FSGS. We then further discuss the potential associations between the excessive secretion of GH and glomerular sclerosis.

## Case presentation

A 63-year-old man was admitted with a two-year history of lower extremity edema. He was diagnosed as nephrotic syndrome, with serum albumin 24 g/L, serum creatinine 46 μmol/L, and urinary protein 4.4 g/24 h. He received a kidney biopsy and the histopathological examinations indicated minimal change disease. Prednisone 1 mg/kg was prescribed for 9 months but led to no response. Then he was admitted to our hospital. His medical history was significant for uncontrolled hypertension for 4 years, diabetes mellitus for 1 years, coronary heart disease for 2 years, and obstructive sleep apnea for more than 10 years, and he was treated with total hip arthroplasty due to osteoarthritis one-year ago.

On admission, physical examination revealed a blood pressure of 135/70 mmHg, temperature 36.4 °C, heart rate 78 /min, respiratory rate 18 /min, weight 105 kg, height 190 cm, body mass index 29 kg/m^2^. He was normal on lung and cardiac auscultation, and had severe bilateral lower extremity edema.

Laboratory test revealed urinary protein excretion of 9.1 g/24 h. Urinalysis showed RBC 2–4/HP. His hemoglobin was 107 (130–175) g/L. His serum albumin was 22.7 g/L, serum creatinine was 130.4 (44–133) μmol/L, and estimated glomerular filtration rate (eGFR) was 49.8 ml/min/1.73m^2^ (CKD-EPI equation). Serum C3 and C4 levels were in normal range. Anti-neutrophil cytoplasmic antibodies, anti-nuclear antibodies, anti-phospholipase A2 receptor antibody were all negative.

**Fig. 1:**
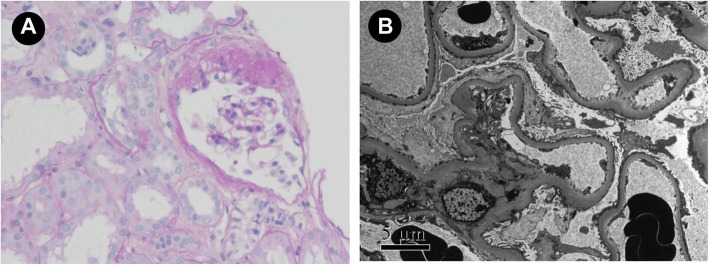
Pathological findings of the kidney biopsy specimens. **a** Light microscopy showed FSGS with acute tubular injury (periodic acid-schiff staining, ×200). **b** Electron microscopy revealed diffuse podocyte foot process effacement (× 5000)

The patient underwent a second kidney biopsy (Fig. 1). Immunofluorescence staining was negative for IgG, IgA, IgM, C3, C1q, or fibrinogen. The specimen for light microscopy contained 17 glomeruli. Three of them showed segmental sclerosis, others showed unremarkable change. Tubular epithelial cells revealed cytoplasmic vacuolation and diffuse flattening. There is scarce interstitial infiltration by mononuclear cells and lymphocytes, along with focal interstitial fibrosis. Electron microscopy revealed diffuse effacement of foot process of podocytes. Thus, the patient was diagnosed as FSGS, not otherwise specified (NOS) variant, with acute tubular injury.

**Fig. 2:**
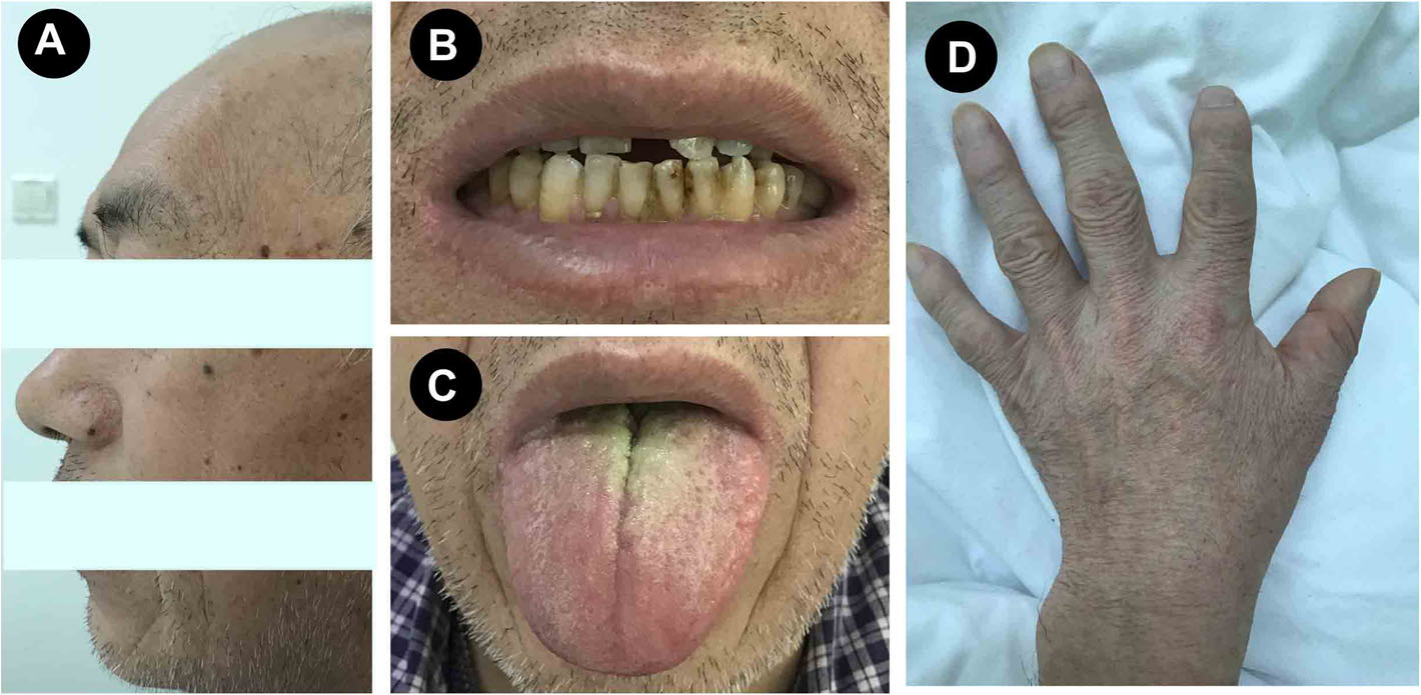
The phenotype led to suspicion of acromegaly. **a** The facial features were coarse, with prognathism, and prominent brow ridges. **b** Wide spacing of the teeth **(c)** Macroglossia. **d** Enlarged hands

**Fig. 3:**
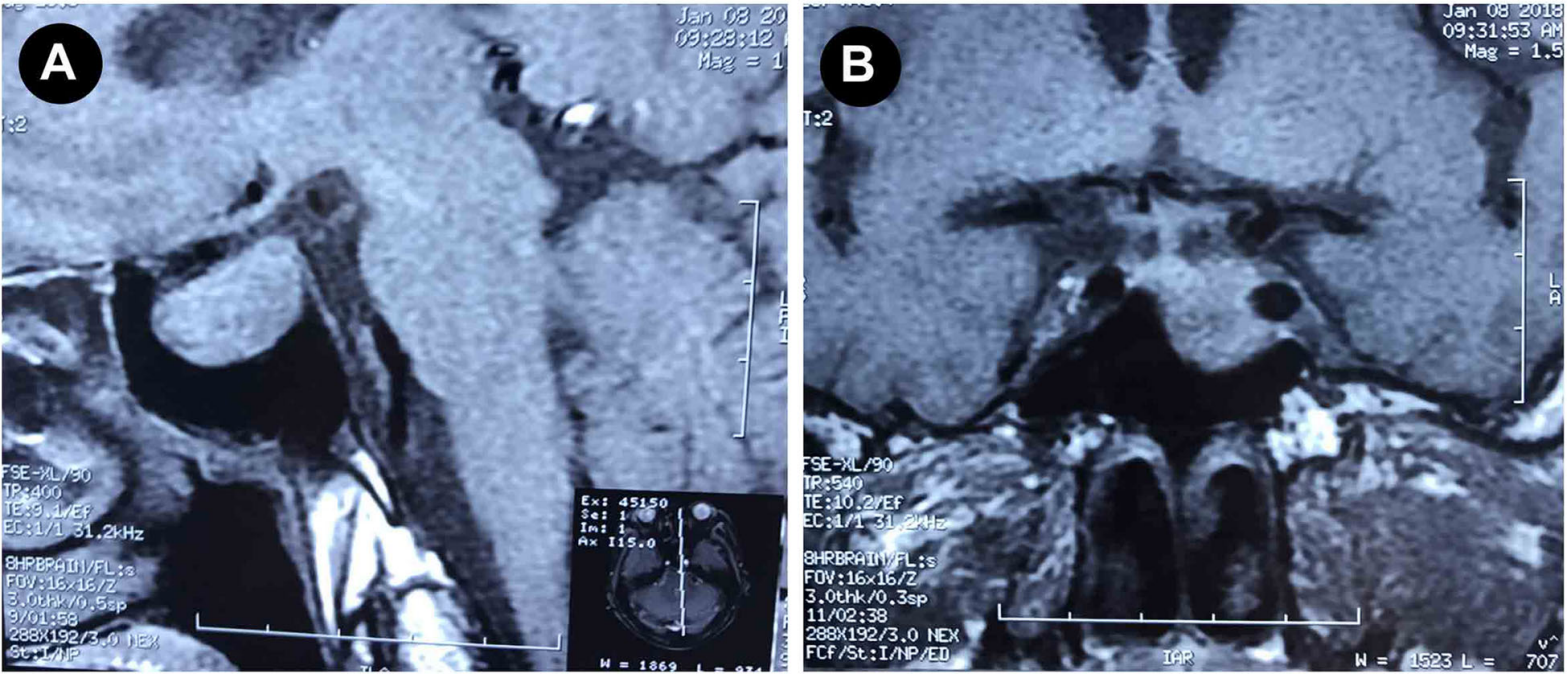
Pituitary magnetic resonance imaging (MRI) revealed a pituitary macroadenoma

In addition, his acromegalic features were noticed and he complained with a long history of soft tissue swelling of hands and feet for over 20 years. His facial features were coarse, with thick lips, prognathism and macroglossia, wide spacing of the teeth with upper incisors spreading apart, prominent brow ridges, and deep voice. The patient reported an enlargement of hands and feet with shoe size increased from 39 to 42 (Fig. 2). He complained with increasing lethargy over the past 20 years and his wife reported his snore at sleep. His visual acuity was 0.6 for left eye and 0.4 for right eye (uncorrected), without visual field defects. We further detected serum GH and IGF-1 levels, and found significantly elevated concentrations of random plasma GH as 39.74 (0.03–2.47) ng/mL and IGF-1 as 998 (75–212) ng/mL. An oral glucose tolerance test (OGTT) was performed and showed failure suppression of GH after a glucose load (fasting concentration of GH was 25.1 ng/mL, which rise to > 50 ng/mL at 120 min after a 75-g oral glucose load) (Table 1). Then, the diagnosis of acromegaly was confirmed by the presence of a pituitary macro-adenoma with a diameter of 1.4 cm in size by a pituitary magnetic resonance imaging (MRI) (Fig. 3).

**Table 1:** Failure suppression of growth hormone after a 75-g glucose load

	0 min	30 min	60 min	120 min
Glucose (mmol/L)	6.7	11.8	13.2	15.7
Growth hormone (ng/mL)	25.7	32.6	> 50	> 50

The tumor was subtotally removed by trans-sphenoidal surgery. Minimal residual tumor encircled the internal carotid artery within the cavernous sinus was reserved, due to the high risk of bleeding. The pathological diagnosis was pituitary adenoma with Ki67 of 1%. Random GH level decreased to the concentration of 5.3 ng/mL and 2.1 ng/mL at one-month and six-month postoperatively, with normal thyroid hormone, adrenocorticotropic hormone (ACTH) and sex hormone levels. During follow-up, he reported that his soft-tissue swelling, snoring and sleep apnea were significantly attenuated, with normalized blood glucose and better-controlled hypertension. Three-month after surgery, his urinary protein excretion was decreased (5.5 g/24 h) and serum albumin was improved (33.3 g/L). Partial remission of nephrotic syndrome was achieved at six-month postoperatively, with urinary protein excretion 2.0 g/24 h, serum albumin 35.0 g/L, normal serum creatinine (80.0 μmol/L) and eGFR (90.4 ml/min/1.73m^2^). The remission was maintained in the follow-up accompanied with the gradual reduce of corticosteroid with oral prednisone.

## Discussion and conclusions

Here, we presented a patient with FSGS in combination with acromegaly. Whether there is any cause-and-effect relationship between FSGS and acromegaly are obscure. The patient was diagnosed as primary FSGS based on the pathological presentation of diffuse podocyte foot process effacement and lack of glomerular enlargement. However, the relation between FSGS and acromegaly could also be viewed that the overproduction of GH with acromegaly was responsible for the development and progress of glomerulosclerosis. Before admission, the patient had received prednisone 1 mg/kg for 9 months but showed no response. After the surgically partial removal of pituitary adenoma, the patient achieved partial remission of proteinuria and maintained it in the follow-up with gradual reduce of corticosteroid. This disease course supported the relationship between acromegaly and FSGS.

So far with our knowledge, there are only two cases reported as glomerulosclerosis associated with acromegaly. In 1999, Yoshida et al. [8] first reported a 46-year-old man with acromegaly followed by FSGS. Therapy with corticosteroid resulted in a partial remission. However, frequent relapses were occurred after dosage reduction. Octreotide acetate was subcutaneously injected, and the pituitary adenoma was subsequently removed by trans-sphenoidal surgery. This led to normalized creatinine clearance, and then steroid dosage was gradually reduced, which was finally maintained remission. The case suggests that GH hypersecretion can participate in renal glomerular diseases progression and development. Takai et al. reported a second case [9] that a 53-year old male patient with moderate proteinuria for 6 years, who was diagnosed with acromegaly for more than 15 years. The renal biopsy revealed pathological manifestations with glomerular hypertrophy and FSGS. However, proteinuria was continued after trans-sphenoidal microsurgery of the adenoma with normalized GH and IGF-1 levels. These two cases proposed that acromegaly may participate, at least in part, in FSGS development and progression. However, the prognosis of glomerulonephritis after acromegaly treatment is variable, which may be related to the course of acromegaly and the severity of glomerulonephritis.

Compelling evidence has proven that GH and IGF-1 secretion participate in physiologically renal function and growth [10]. GH receptors are indispensable for the direct role of GH in kidney [11]. The kidneys of adult human can express GH receptor, IGF-1, IGF-1 receptor, and IGF-1 binding proteins [11]. Moreover, these proteins are diversely expressed in different segments of the nephron with variable anatomy and function. Thus, it indicates that GH and IGF-1 secretion may play extensive roles in different nephron [12]. The patients with acromegaly often showed renal hypertrophy with increased kidney diameter as compared to controls [13]. In addition, they show significantly increased renal plasma flow and glomerular filtration [14]. One recent study showed that eGFR was increased by 25% in patients with active acromegaly and remission with surgical can lead to eGFR reduction [15]. However, evidence about renal architecture in patients diagnosed with acromegaly is scarce, due to renal biopsy limitation. The three reported cases [8, 9] (including the current one) all presented with FSGS in the kidneys with or without glomerular hypertrophy. Using a transgenic mouse models overexpressing GH genes, Palmiter et al. [16] found that the weight of kidneys were larger in GH transgenic mice, and mesangial proliferation and glomerular hypertrophy were developed at 4 to 5 week-old GH transgenic mice. At 19 weeks of age, it showed progressive mesangial sclerosis, and at 30 to 37 weeks of age, the mice indicated complete glomerulosclerosis. Similarly, it showed that rats with acromegaly exhibited as 3.5 times large as renal hypertrophy and severe glomerulosclerosis [17]. In addition, Trachtman H et al. found a deleterious effect of GH on glomerular structure in an animal model of FSGS [18]. All these evidences support that overproduction of GH and IGF-1 in acromegaly is associated with glomerular sclerosis and hypertrophy [19].

The current patient had severe pituitary adenoma of a large size with cystic degeneration and necrosis which indicate pituitary apoplexy. Moreover, He was accompanied with several comorbidities associated with acromegaly, including obstructive sleep apnea and hip arthroplasty due to osteoarthritis, and coronary heart disease, diabetes mellitus, and hypertension [20–22]. His left cavernous sinus was surrounded and partially invaded by the tumor. Thus, he received subtotally removal of the pituitary adenoma. The partial remission of proteinuria may be due to the long-term course of acromegaly and its permanent effects on FSGS, and possibly not-complete removal of pituitary adenoma.

In conclusion, this rare case might suggest a relationship between FSGS and acromegaly. We speculate the scarcity of the association between excessive GH and glomerular sclerosis may be due to lack of awareness, lower GH levels in patients with acromegaly versus experimental animals, and low-grade proteinuria in most patients versus nephrotic syndrome in this case. Acromegaly should be considered in patients with FSGS who present with the clinical features of GH excess. An early diagnosis and treatment of acromegaly may be beneficial to the patients with FSGS.

## Abbreviations

ACTH: Adrenocorticotropic hormone
eGFR: Estimated glomerular filtration rate
FSGS: Focal segmental glomerulosclerosis
GH: Growth hormone; GHRs: GH receptors
IGF-1: Insulin-like growth factor 1
MRI: Magnetic resonance imaging
NOS: Not otherwise specified
OGTT: Oral glucose tolerance test;
